# A review on anti-cancer properties of quercetin in gastric cancer

**DOI:** 10.3389/fphar.2025.1563229

**Published:** 2025-05-19

**Authors:** Xiaoxia Xie, Yan Wei

**Affiliations:** Department of pharmacy, 970 Hospital of the PLA Joint Logistic Support Force, Yantai, Shandong, China

**Keywords:** quercetin, gastric cancer, anti-proliferation, cell death, anti-inflammatory

## Abstract

This review paper focuses on the multifaceted roles and therapeutic potential of quercetin in gastric cancer (GC). Quercetin, a natural flavonoid compound abundant in dietary sources such as nuts, teas, vegetables, and herbs, has garnered significant attention due to its anticancer properties. Accumulated evidence demonstrates quercetin’s inhibitory effects against GC by targeting key pathways such as cell cycle regulation, fatty acid synthesis, and mitochondrial apoptosis, thereby exerting anti-proliferative and apoptotic strategies. Furthermore, quercetin effectively alleviates GC-related inflammation, optimizing the tumor microenvironment. Mechanistically, quercetin induces various forms of programmed cell death in GC cells, including ferroptosis, pyroptosis, and autophagy, through regulating specific molecular targets. Additionally, quercetin inhibits GC angiogenesis by downregulating vascular endothelial growth factor A (VEGF-A) and its receptor VEGFR-2 expression, and demonstrates anti-metastatic effects by modulating urokinase-type plasminogen activator (uPA) and its receptor (uPAR) function. As research delves deeper into the mechanisms of quercetin’s actions and its validation in clinical trials, its prospects as a novel therapeutic agent for GC become increasingly promising. Quercetin’s low toxicity and ability to synergize with other anticancer drugs make it a potential key component in comprehensive GC treatment strategies, significantly enhancing patient prognosis and quality of life.

## 1 Introduction

Gastric cancer (GC) ranks as the fifth most common malignancy globally and is the fourth leading cause of cancer-related deaths, with an estimated 1.08 million new cases and 769,000 fatalities reported in 2020 alone (Smyth et al., 2020; Sung et al., 2021). This high mortality is exacerbated by regional disparities: over 70% of cases occur in Eastern Asia, particularly in China, Japan, and Korea, where dietary habits (e.g., high salt intake, smoked foods), *Helicobacter pylori* infection, smoking, and genetic predisposition synergistically drive carcinogenesis (Machlowska et al., 2020; Thrift and El-Serag, 2020).

Current treatment strategies for GC remain suboptimal. While endoscopic resection and gastrectomy are curative for early-stage tumors (Sexton et al., 2020), locally advanced or metastatic GC relies on chemotherapy (e.g., fluoropyrimidines, platinum agents) and targeted therapies (e.g., trastuzumab) (Zeng and Jin, 2022). However, these approaches are plagued by severe toxicities—neuropathy, myelosuppression, and mucositis—and intrinsic/acquired drug resistance. Consequently, there is an urgent need for safer, multi-targeted agents that circumvent these limitations.

**FIGURE 1:**
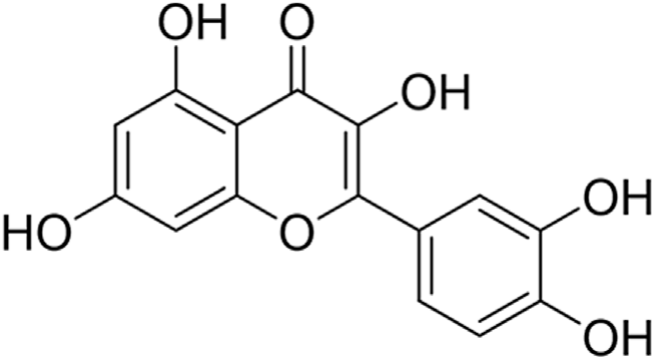
The structural formula for quercetin.

Natural compounds, particularly flavonoids, have emerged as promising candidates due to their low toxicity and pleiotropic mechanisms (Xu et al., 2022; Dai et al., 2024). Quercetin (Figure 1), a flavonoid ubiquitously found in dietary sources such as nuts, teas, vegetables, and herbs, has garnered significant attention from the scientific community due to its multifaceted biological activities (Hosseini et al., 2021; Di Petrillo et al., 2022; Alizadeh and Ebrahimzadeh, 2022). This bioactive compound exists predominantly in two forms: the free aglycone state and as derivatives conjugated with molecules like carbohydrates, lipids, alcohols, and sulfate groups, resulting in compounds like quercetin glycosides, prenylated quercetin, quercetin ethers, and quercetin sulfates. This structural diversity not only enhances quercetin’s solubility and stability but also modulates its biological effects.

Extensive research has delved into the mechanisms underpinning quercetin’s anticancer potential, uncovering multiple pathways that synergistically contribute to its therapeutic outcomes (Reyes-Farias and Carrasco-Pozo, 2019; Boretti, 2023). Accumulated evidence demonstrates quercetin’s inhibitory effects against a diverse range of cancer cells, encompassing breast, lung, nasopharyngeal, kidney, colorectal, prostate, pancreatic, and ovarian malignancies (Shafabakhsh and Asemi, 2019; Khorsandi et al., 2017; Guo et al., 2021; Li and Li, 2023; Lugano et al., 2020; Neamtu et al., 2022). These discoveries suggest that quercetin’s antioxidant, anti-inflammatory, and antiproliferative properties may converge to target cancer cells, disrupting their growth and progression. This review article focuses on summarizing the mechanistic insights into quercetin’s role in gastric cancer, thereby providing theoretical underpinnings for its potential in the fight against this devastating disease.

## 2 Sources and metabolism of quercetin

Quercetin is a natural flavonoid compound with a molecular structure characterized by multiple ring structures and a hydroxyl group, specifically comprising three carbon atom frameworks of C6-C3-C6 and a glucose unit attached to a hydroxyl group. This unique molecular structure endows quercetin with potent biological activity. Quercetin has a wide range of sources and is primarily found in the leaves, fruits, and roots of plants, such as onions, shallots, asparagus, cabbage, grapefruit, and various other foods, as well as in medicinal plants like sophora flower buds and Chinese arborvitae leaves (Di Petrillo et al., 2022).

The metabolism of quercetin primarily occurs in organs such as the small intestine, colon, liver, and kidneys (Ulusoy and Sanlier, 2020). Upon ingestion, quercetin interacts with proline-rich salivary proteins in the mouth through hydrogen bonds or hydrophobic interactions, forming soluble quercetin-protein aggregates, which do not interfere with its absorption. In the stomach, only a small amount of quercetin is degraded into phenolic acids. In the small intestine, quercetin glycosides (QGs) are completely absorbed by intestinal epithelial cells via sodium-dependent glucose cotransporters (SGLTs) and then hydrolyzed to quercetin aglycone by intracellular β-glucosidase (CBG) (Ader et al., 2001). Alternatively, some quercetin glycosides can be deglycosylated to quercetin by lactase-phlorizin hydrolase (LPH) (Day et al., 2003). Subsequently, these quercetins and their metabolites undergo extensive phase II metabolism in the liver and small intestine, including glucuronidation, sulfation, and methylation, resulting in derivatives such as quercetin-3-glucuronide, quercetin-3′-sulfate, and 3′-O-methylquercetin (isorhamnetin) (Terao et al., 2008). Some of these metabolites enter the bile and circulatory system via multidrug resistance-associated proteins (MRPs) and organic anion transporters (OATs), ultimately being excreted in urine and feces. Notably, unabsorbed quercetin and its metabolites are also eliminated with feces. Additionally, intestinal microbiota plays a crucial role in quercetin metabolism by degrading it into more easily absorbable low-molecular-weight phenolic compounds, such as 3,4-dihydroxyphenylacetic acid and 4-hydroxybenzoic acid (Tang et al., 2016).

## 3 Application of quercetin in the treatment of gastric cancer

### 3.1 Anti-proliferation and induce apoptosis

Recent studies highlight the promising role of quercetin, a plant-derived flavonoid, in triggering apoptosis in gastric cancer cells. Multiple studies have shown that quercetin effectively inhibits the proliferation of gastric cancer cells and induces their apoptosis through various mechanisms.

Firstly, quercetin significantly impacts the G1 phase of gastric cancer cells by inhibiting the expression of genes such as cyclin D1, P21, and Twist, thereby inhibiting cell proliferation. In particular, Quercetin effectively inhibits the expression of Twist through the P38 mitogen-activated protein kinase (P38MAPK) pathway, reducing the phosphorylation of P38MAPK, a hallmark event in cell proliferation, and thus exerts an anti-proliferative effect (Zhang et al., 2017).

Reactive oxygen species (ROS) are considered as tumor inhibitors, which can affect cell survival and death by affecting mitochondrial function and regulating B-cell lymphoma-2 (Bcl-2) family proteins. Quercetin has been found to induce apoptosis in gastric cancer cells by regulating mitochondrial membrane potential and increasing the production of ROS. It decreases the expression of anti-apoptotic proteins such as myeloid cell leukemia-1(Mcl-1), Bcl-2, and B-cell lymphoma-x (Bcl-x) while increasing the expression of pro-apoptotic proteins such as Bcl-2 associated agonist of cell death (Bad), Bcl-2-Associated x (Bax), and BH3 interacting domain death agonist (Bid), thereby promoting apoptosis (Shang et al., 2018; Wang et al., 2012). These changes significantly affect the survival status of gastric cancer cells by influencing the mitochondrial apoptosis pathway and the death receptor pathway.

It is worth noting that the combined application of quercetin with other anticancer drugs has also shown promising therapeutic effects. For example, when combined with irinotecan or its metabolite SN-38, quercetin can more effectively reduce the metastatic and invasive capabilities of gastric cancer cells and inhibit the epithelial-mesenchymal transition (EMT) process by downregulating the expression of β-catenin, Integrin-β6 (ITG-β6), and Twist Family BHLH Transcription Factor 1 (Twist-1) (Lei et al., 2018). Additionally, the combined treatment of quercetin with siRNA targeting Cell-division cycle protein 20 (CDC20) has demonstrated potential in gastric cancer therapy by downregulating CDC20 expression and significantly inhibiting the growth of gastric cancer cells (Hemati et al., 2019).

### 3.2 Ferroptosis

Ferroptosis is a form of programmed cell death caused by lipid peroxidation, which is regulated by abnormalities in the antioxidant system and iron metabolism. The study found that quercetin significantly increased lipid peroxidation levels in GC cells, and transmission electron microscopy confirmed an increase in ferroptosis in quercetin -induced GC cells (Ding et al., 2024). Further mechanistic exploration revealed that quercetin binds to a sodium-dependent glutamine transporter -Solute carrier family 1 member 5 (SLC1A5) at residues SER-343, SER-345, ILE-423, and THR-460, inhibiting its expression. This binding blocks the nuclear translocation of nuclear factor erythroid 2-related factor 2 (NRF2), resulting in downregulated expression of cystine/glutamate antiporter (xCT)/Glutathione Peroxidase 4 (GPX4) and subsequently weakened antioxidant capacity of the cells. Meanwhile, the quercetin/SLC1A5 signal activates the p-Calcium-calmodulin (CaM)-dependent protein kinase II (p-Camk2)/p-Dynamin-related protein 1 (p-DRP1) pathway, promoting the release of ROS. Additionally, quercetin increases intracellular iron content by inhibiting SLC1A5. These three changes collectively lead to ferroptosis in GC cells.

### 3.3 Pyroptosis

Pyroptosis, as a unique form of cell death, is characterized by the rapid rupture of the cell membrane and the release of intracellular pro-inflammatory contents. The core of this process lies in the activation of the gasdermin family of proteins, particularly the cleavage and oligomerization of Gasdermin D (GSDMD). In the latest research on GC cells, quercetin has demonstrated its powerful potential to induce pyroptosis in GC cells (Rong et al., 2023).

Using Western blot analysis, researchers observed significant alterations in the expression of a series of proteins tightly associated with pyroptosis in GC cells treated with quercetin. Notably, the expression levels of pyroptosis markers such as GSDMD, Gasdermin-E (GSDME), cleaved cysteinyl aspartate specific proteinase 1 (C-CASP1), and NOD-like receptor protein 3 (NLRP3) exhibited a pronounced upregulation trend. This upregulation not only confirms the direct activation of the pyroptotic pathway in GC cells by quercetin but also further reveals a positive correlation between the intensity of its effect and the concentration of quercetin. In other words, as the concentration of quercetin gradually increases, the expression levels of pyroptosis-related proteins in GC cells also enhance, thereby more vigorously promoting the pyroptotic process in these cells.

### 3.4 Autophagy

Autophagy, an evolutionarily conserved, dynamic, and lysosome-mediated process, involves the sequestration and delivery of cytoplasmic materials to lysosomes for degradation and recycling. In the context of cancer, autophagy plays a complex and multifaceted role. On one hand, it serves as a tumor suppressor mechanism by eliminating potential oncogenic factors and damaged organelles, thereby maintaining cellular homeostasis and inhibiting tumor initiation. On the other hand, in established tumors, autophagy can be exploited by cancer cells to cope with nutritional deprivation, hypoxia, or therapeutic stress, promoting cancer cell survival and adaptation, thus accelerating tumor progression and the development of drug resistance.

Among the natural compounds being explored for cancer therapy, quercetin has garnered significant attention for its ability to trigger autophagy in human gastric cancer cells. Functional analyses have revealed that inhibition of autophagy significantly enhances quercetin-induced apoptosis in gastric cancer cells, suggesting that quercetin-induced autophagy acts as a protective mechanism in these cells (Wang et al., 2011). Quercetin activates autophagy by suppressing the Akt-mTOR signaling cascade. This involves dephosphorylating Akt and mTOR, which in turn inactivates downstream effectors such as p70 S6K and 4E-BP1.Furthermore, quercetin stabilizes hypoxia-inducible factor-1α (HIF-1α), a transcription factor that regulates critical biological processes such as cell survival, metabolism, and invasion. The accumulation of HIF-1α not only inhibits the mTOR signaling pathway but also enhances autophagy by upregulating the expression of BCL2 and adenovirus E1B 19-kDa-interacting protein 3 (BNIP3)/BNIP3-like (BNIP3L), which disrupts the interaction between Beclin 1 and Bcl-2/Bcl-xL, thereby facilitating autophagy.

In other oncogenic studies, quercetin has been shown to trigger excessive autophagy in tumor cells, ultimately leading to cell death (Estrada-Villaseñor et al., 2021; Psahoulia et al., 2007). This dual role of quercetin in modulating autophagy—demonstrating both pro-survival and pro-death effects—may arise from contextual differences in intracellular microenvironments, as well as variations in drug exposure time and concentration.

### 3.5 Metastasis and angiogenesis

Angiogenesis, a crucial process in tumor growth and metastasis, supplies essential oxygen and nutrients to tumors, thereby promoting their proliferation and distant spread. In the context of GC, angiogenesis is particularly pivotal. Research has demonstrated that quercetin significantly inhibits GC-related angiogenesis (Lei et al., 2018). Specifically, quercetin downregulates the expression of Vascular Endothelial Growth Factor A (VEGF-A) and its receptor- Vascular Endothelial Growth Factor receptor-2 (VEGFR-2) in GC cells and mouse models. The VEGF-A/VEGFR-2 signaling pathway, being one of the primary regulators of angiogenesis, is inhibited by quercetin, thereby suppressing GC angiogenesis. Furthermore, in the AGS mouse model, when quercetin is combined with low-dose irinotecan, a notable reduction in the proportion of Tie2^+^ monocytes is observed. As Tie2^+^ monocytes are intimately associated with angiogenesis, their decrease likely contributes further to the inhibition of GC angiogenesis.

Moreover, the metastasis and invasion of GC cells are major factors contributing to poor patient prognosis. Quercetin inhibits these abilities of GC cells through multiple mechanisms (Li and Chen, 2018). The urokinase-type plasminogen activator (uPA) and its receptor (uPAR) play vital roles in GC metastasis. Quercetin significantly reduces the activity and expression levels of uPA and uPAR in GC cells, consequently inhibiting their migration and invasion. When quercetin is used in conjunction with uPAR knockdown, it inhibits the enzymatic activity of matrix metalloproteinase-2 (MMP-2) and matrix metalloproteinase-9 (MMP-9), effectively blocking the Pak1-Limk1-cofilin signaling pathway and suppressing the invasive capabilities of GC cells. Additionally, quercetin downregulates the expression of EMT (epithelial-mesenchymal transition)-related genes such as ITG-β6 and Twist-1 in GC cells, which are crucial in the process of tumor metastasis and invasion.

### 3.6 Anti-inflammatory

Gastric cancer is often accompanied by inflammatory reactions, which play a pivotal role in its initiation and progression. For instance, inflammatory factors such as tumor necrosis factor-α (TNF-α) in gastric cancer can induce the expression of MMP-9, facilitating the migration and invasion of tumor cells (Hsieh et al., 2022). Furthermore, *Helicobacter pylori* (HP) infection, the primary cause of gastritis, exacerbates the risk of gastric cancer by triggering inflammatory responses in the gastric mucosa.

Quercetin exhibits remarkable anti-inflammatory effects. In the context of gastric cancer-related inflammation, quercetin inhibits TNF-α-induced MMP-9 expression by downregulating the expression of inflammation-related genes and proteins through the TNF-α -Src family kinase c (c-Src)- Extracellular-regulated kinase 1/2 (ERK1/2) and c-fos proto-oncogene protein (c-Fos) or nuclear factor kappa-B (NF-κB) pathways. Additionally, quercetin alleviates apoptosis and inflammatory damage in gastric epithelial cells induced by HP infection by inhibiting the Sp1 Transcription Factor (SP1)/Lipocalin 2 (LCN2) axis. Collectively, these mechanisms underscore the crucial anti-inflammatory and protective roles of quercetin in gastric cancer-associated inflammation.

## 4 Application of quercetin preparations

In recent years, nanomedicines have been widely applied in various therapeutic fields, particularly in oncology, where their use has significantly enhanced the safety and efficacy of common anticancer drugs. The primary advantages of nanomedicines and nanotechnology-based delivery systems lie in effective targeting, delayed release, prolonged half-life, and reduced systemic toxicity. To overcome these limitations and enhance the biodistribution of quercetin after administration, nanotechnology-based drug delivery systems have been developed. Numerous studies have indicated that, compared to its free form, the nanoformulation of quercetin exhibits more pronounced anticancer effects. Additionally, incorporating quercetin into various nano-delivery systems improves its sustained release and stability, extends its circulation time, enhances its accumulation at target sites, and boosts its therapeutic efficiency (Zhu et al., 2017; Ghanbari-Movahed et al., 2022).

Gao’s research group has crafted a hyaluronic acid (HA)-modified silica nanoparticle system, termed HA-SiLN/QD, designed to concurrently deliver quercetin and doxorubicin (DOX) for augmenting GC treatment outcomes (Sun et al., 2022). This innovative system leverages quercetin’s ability to downregulate Wnt16 and P-glycoprotein expression, thereby modifying the tumor microenvironment and reversing multidrug resistance (MDR) to bolster DOX’s effectiveness. In detail, quercetin plays a pivotal role in reducing Wnt16 and P-gp levels, further facilitating tumor microenvironment remodeling and MDR reversal to enhance DOX’s functionality. The nanoscale HA-SiLN/QD boasts remarkable stability and sustained release characteristics. *In vitro* tests on SGC7901/ADR cells revealed targeted uptake through HA-facilitated endocytosis, and *in vivo* anticancer evaluations in SGC7901/ADR tumor-bearing mice models distinctly demonstrated HA-SiLN/QD’s ability to inhibit tumor growth.

## 5 Discussion and conclusion

**FIGURE 2:**
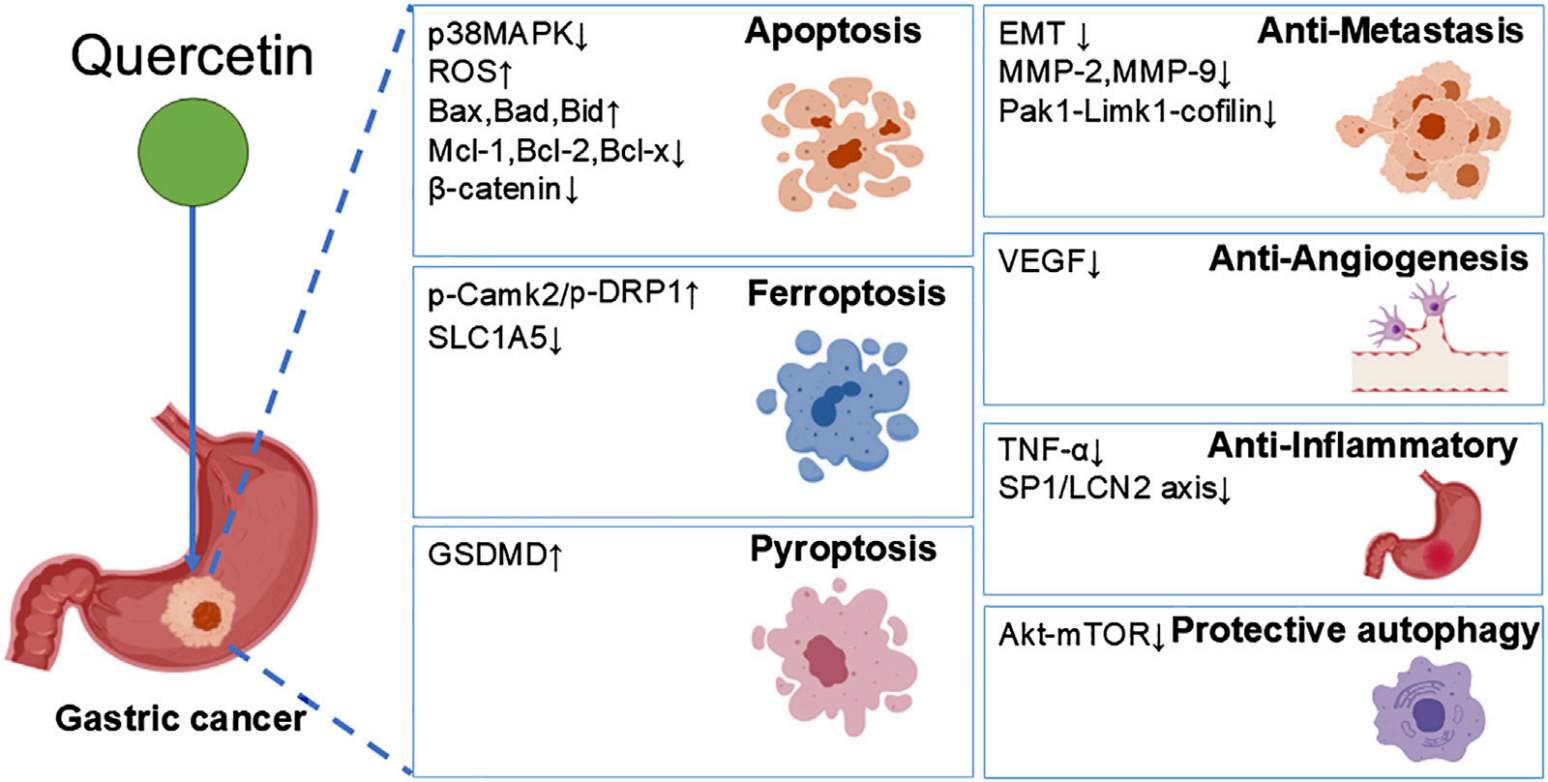
Quercetin exerts dual effects on GC cells. On one hand, it suppresses GC progression through induction of apoptosis, pyroptosis, ferroptosis, as well as inhibition of migration, angiogenesis, and inflammation; on the other hand, it promotes GC cell survival by triggering protective autophagy.

This review comprehensively summarizes the multifaceted roles and potential therapeutic applications of quercetin in gastric cancer (Table 1; Figure 2). Quercetin precisely targets key pathways such as cell cycle, fatty acid synthesis, and mitochondrial apoptosis, effectively implementing anti-proliferative and apoptotic strategies. Its uniqueness lies in its ability to induce ferroptosis and pyroptosis, two novel forms of programmed cell death, thereby opening up new avenues for GC treatment. Additionally, quercetin significantly inhibits angiogenesis and metastasis in GC cells, directly targeting the crucial aspects of tumor progression. With its anti-inflammatory properties, quercetin effectively alleviates GC-related inflammation, optimizing the tumor microenvironment.

**TABLE 1:** Mechanism of quercetin in the inhibition of gastric cancer. Mechanism of quercetin in the inhibition of gastric cancer.

*In vitro* (concentration)	*In vivo* (concentration)	Main pharmacological effects	Mechanisms	Refs
GES-1 cells (33 μM)	H. pylori-Infected Mouse Model (64 mg/kg, P.O, 72 h)	Anti-proliferation and induce apoptosis	Inhibiting the expression of genes such as cyclin D1, P21, and Twist	Zhang et al. (2017)
AGS (10–320 μM); BGC-823 (15–120 μM); SGC-7901, MKN45, EC109, SW116, GES-1 cells (30–120 μM)			Production of ROS,decreases the expression of anti-apoptotic proteins such as Mcl-1, Bcl-2, and Bcl-x while increasing the expression of pro-apoptotic proteins such as Bad, Bax, and Bid	Shang et al. (2018), Wang et al. (2012)
AGS cells (6.25–100 μM)	Xenograft tumor model of AGS cell line. (20 mg/kg, i.v, 3 times a week, 4 weeks)		Inhibit the EMT process by downregulating the expression of β-catenin, ITG-β6, and Twist-1	Lei et al. (2018)
AGS cells (25–413 μM)			Downregulating CDC20 expression and significantly inhibiting the growth of gastric cancer cells	(Hemati et al., 2019) (Ding et al., 2024)
GC cell lines AGS (CL-0022) (IC_50_: 38 μM), HGC-27 (CL-0107), MKN-7 (CL-0574) MKN-45 (CL-0292), SNU-1 (IC_50_: 40 μM) (CL-0474), and NCI–N87 (CL-0169) GES-1 (GGCC-002-0010) (2.5–160 μM)	Four-week-old female BALB/c nude mice 20 mg/kg, i.v, 3 times a week, 4 weeks	Ferroptosis	Quer promotes ferroptosis in GC cells and suppresses GC progression	Rong et al. (2023)
AGS cells (20–80 μM)		Pyroptosis	Activation of the gasdermin family of proteins	Wang et al. (2011)
AGS (IC_50_: 40 μM) and MKN28 (IC_50_: 160 μM) cells (3.125–200 μM)	Tumor xenograft model 50 mg/kg, i.v,/every day	Autophagy	Inhibition of the Akt-mTOR signaling pathway	Estrada-Villaseñor et al. (2021)
AGS cells (6.25–100 μM)	Xenograft tumor model of AGS cell line. 20 mg/kg i.v, 3 times a week/4 weeks	Metastasis and angiogenesis	Inhibits GC-related angiogenesis	Lei et al. (2018)
MGC803, GC7901, BGC823 10 μM AGS 10 μM, and N87, GES-1			Reduces the activity and expression levels of uPA and uPAR in GC cells	Psahoulia et al. (2007)
GES-1 cells (0.01–100 μM)		Anti-inflammatory	Inhibits TNF-α-induced MMP-9 expression by downregulating the expression of inflammation-related genes and proteins through the TNFR-c-Src–ERK1/2 and c-Fos or NF-κB pathways	Li and Chen (2018)

While the preclinical evidence supporting quercetin’s anti-GC effects is compelling, several limitations and inconsistencies in existing studies warrant critical discussion. First, many mechanistic studies rely heavily on *in vitro* models, which may not fully recapitulate the complexity of the tumor microenvironment *in vivo*. For instance, variations in quercetin’s efficacy across different GC cell lines highlight potential cell-type-specific responses, yet the underlying reasons for these discrepancies remain underexplore (Shang et al., 2018; Wang et al., 2012; Lei et al., 2018). Additionally, the concentrations of quercetin used in *in vitro* experiments (often exceeding 50 μM) may not reflect physiologically achievable levels in humans due to its rapid metabolism and poor bioavailability (Alizadeh and Ebrahimzadeh, 2022).

While quercetin’s multi-targeted effects, such as ferroptosis, pyroptosis, and autophagy are well-documented, conflicting results exist regarding its role in autophagy. Some studies suggest that quercetin-induced autophagy acts as a cytoprotective mechanism in GC cells, while others report its pro-death effects in other cancers. This dichotomy underscores the context-dependent nature of autophagy and necessitates further investigation into the molecular switches.

**FIGURE 3:**
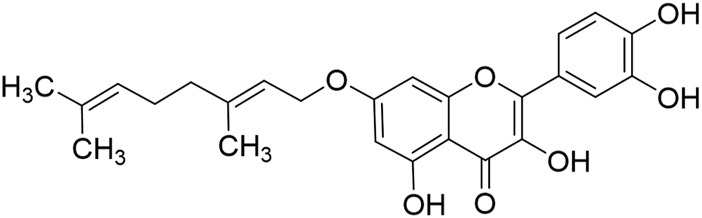
The structural formula for 7-O-Geranylquercetin.

The low solubility in water, poor bioavailability, and rapid clearance, metabolism, and enzymatic degradation of quercetin significantly hinder its potential for clinical application as an anticancer drug. Structural modifications and nanoformulations have improved quercetin’s solubility and tumor-targeting efficiency. For example, Zhen’s research group synthesized 7-O-Geranylquercetin (GQ, Figure 3), a monoalkyl derivative of quercetin with enhanced lipid solubility, which demonstrated superior inhibitory effects on the growth of human gastric cancer cell lines SGC-7901 and MGC-803 compared to quercetin, while showing no significant toxicity to human gastric cells (GES-1 cells), indicating that structural modifications effectively improve quercetin’s activity in gastric cancer treatment (Fang et al., 2018). However, the long-term safety of synthetic derivatives and nanocarriers remains to be experimentally evaluated to avoid unintended toxicity. Furthermore, most studies focus on monotherapy, whereas quercetin’s synergistic effects with chemotherapy (e.g., irinotecan) or targeted therapies need rigorous validation in resistant GC models.

Despite promising preclinical evidence demonstrating quercetin’s therapeutic potential, clinical data validating its efficacy in GC patients remain sparse. Current clinical investigations predominantly evaluate quercetin as a dietary supplement for managing chronic conditions such as diabetes and cardiovascular diseases (Alizadeh and Ebrahimzadeh, 2022), with limited emphasis on its oncological applications. To unlock its anticancer potential, priority should be given to developing innovative drug delivery systems—such as lipid-based nanoparticles and cyclodextrin inclusion complexes—to overcome its poor bioavailability and enhance tumor-targeted accumulation. Furthermore, comprehensive preclinical evaluations of chronic toxicity profiles and drug-interaction potentials are critically needed to establish safe dosing regimens and mitigate risks during clinical translation.

As research delves deeper into the mechanisms of quercetin’s actions and its validation in clinical trials, its prospects as a novel therapeutic agent for GC become increasingly promising. Furthermore, quercetin’s low toxicity and its ability to synergize with other anticancer drugs make it a potential key component in comprehensive GC treatment strategies, significantly enhancing patient prognosis and quality of life.
